# Scarlatina and Variola

**Published:** 1894-12

**Authors:** 


### SCARLATINA AND VARIOLA.

TRANSLATED BY FREDERIC PRESTON, M. D., CHESTER, PA.

The cases observed of eruptive fevers following each other at near intervals are not exceptional; much more rare are those cases where the incubation seems to have been simultaneous. Such seems to have been the case of the patient of M. Wolberg, a little girl of six years and a half, vaccinated, who was attacked by a scarlatina, well characterized and not doubtful, since her brothers and sisters were attacked by it a short time after.

The scarlatina evolved regularly until the sixth day after the appearance of the eruption; fever and malaise showed themselves for the second time. Four days later there appeared, first on the face, and then on the body, an eruption of variola (or of varioloid, to judge by the description of the author), which eruption passed through the stage of desiccation, and the patient rapidly recovered.

If, with the greater number of French authors, we fix three or four days as the period of incubation for scarlatina, and about ten days for that of variola, one sees that the child must have contracted the two diseases at almost the same period.

It is curious to note that each disease proceeded regularly without any apparent reciprocal influence on their respective duration or gravity. Unfortunately, it was impossible to find the origin of this double contagion.—From *La Semaine Médicale,* Paris, 27th October, 1894, p. 488.

